# Study of the Influence of Phase Noise on the MEMS Disk Resonator Gyroscope Interface Circuit

**DOI:** 10.3390/s20195470

**Published:** 2020-09-24

**Authors:** Wenbo Zhang, Weiping Chen, Liang Yin, Xinpeng Di, Dongliang Chen, Qiang Fu, Yufeng Zhang, Xiaowei Liu

**Affiliations:** 1 MEMS Center, Harbin Institute of Technology, Harbin 150000, China; 17b321006@stu.hit.edu.cn (W.Z.); weipingchen.hit@gmail.com (W.C.); 16B321002@hit.edu.cn (D.C.); qiangfu.hit@gmail.com (Q.F.); yufengzhang.hit@gmail.com (Y.Z.); xiaoweiliu.hit@gmail.com (X.L.); 2 Key Laboratory of Micro-Structures Manufacturing (Harbin Institute of Technology), Ministry of Education, Harbin 150000, China; 3 Shanghai Aerospace Control Technology Institute, Shanghai 201109, China; dixinpeng1@163.com; 4 State Key Laboratory of Urban Water Resource & Environment, Harbin Institute of Technology, Harbin 150000, Heilong Jiang Province, China

**Keywords:** disk resonator gyroscope, phase noise, gyroscope interface circuit, bias stability

## Abstract

In this paper, a detailed analysis of the influence of phase noise on the micro-electro-mechanical system (MEMS) disk resonator gyroscope (DRG) is presented. Firstly, a new time-varying phase noise model for the gyroscope is established, which explains how the drive loop circuit noise converts into phase noise. Different from previous works, the time-varying phase noise model in this paper is established in mechanical domain, which gain more physical insight into the origin of the phase noise in gyroscope. Furthermore, the impact of phase noise on DRG is derived, which shows how the phase noise affects angular velocity measurement. The analysis shows that, in MEMS DRG, the phase noise, together with other non-ideal factors such as direct excitation of secondary resonator, may cause a low frequency noise in the output of the gyroscope system and affect the bias stability of the gyroscope. Finally, numerical simulations and experiment tests are designed to prove the theories above.

## 1. Introduction

As a new generation gyroscope, the micro-electro-mechanical system disk resonator gyroscope (MEMS DRG) is an attractive candidate for high-performance MEMS gyroscopes due to its near navigational grade precision and small volume [1,2,3,4,5]. As a solid wave gyroscope (SWG), MEMS DRG inherently possesses a high Q factor and mode match mechanism, which maintain its low mechanical-thermal noise and high mechanical sensitivity [6,7,8,9]. However, due to the inevitable manufacturing imperfections, maintaining high quality factor during the mode matching is by no means trivial. During the last few years, large efforts have been made to improve quality factor and reduce frequency split [10]. Based on the frequency analysis of DRG perturbed by point masses and springs, several successful implementations have been employed to reduce the frequency differences without sacrificing the quality factor [11,12,13]. The high quality factor and mode match mechanisms also reduce the gain requirement in the sense loop, leading to low output electricity noise [14,15]. Therefore, both mechanical-thermal noise and electricity noise are much smaller than traditional vibration gyroscopes. As mechanical and circuit noise are decrease, other noise sources can no longer be ignored.

Phase noise is a concept usually used in high-frequency applications such as wireless communication and MEMS oscillators [16,17,18,19,20]. Several models have been established thus far to predict the phase noise [21,22,23,24,25,26,27,28,29]. Generally, previous approaches may be classified as a linear time-invariant (LTI) model, linear time-variant (LTV) model, and nonlinear time-variant (NLTV) model. As a starting point, the LTI model provides important qualitative design insights but fails to explain the experimental observations of the up-conversion of low-frequency noise [21]. LTV model incorporates the time-varying nature of oscillator and explains quantitatively the mechanism by which noise sources of all types convert to phase noise [22]. However, the LTV model assumes the frequency does not change with the amplitude variation and loses sight of the impact of nonlinear effects. Therefore, LTV model misses an important phase noise source. NLTV model incorporates the nonlinear effects and the analyze method is based on numerical techniques such as state space method proposed by Kaertner and Demir et al. [27]. NLTV model agrees well with the practical case. However, due to the complexity in mathematics, this model shows less physical intuition and can hardly guide the practical design process.

Specially, in micro-electromechanical system such as MEMS Resonator, numerical approaches have made significant progress in recent years [24,30,31,32,33]. Aside from phase noise predicted in LTV model, the main addition term is caused by nonlinear effects in MEMS resonator [34,35]. Nonlinear models of MEMS disk resonators have been exclusively investigated [36,37,38]. Chorsi et al. [36,37] modeled MEMS disk resonators, including the influences of intermolecular forces such as van der Waals and Casimir, and found the intermolecular forces may cause additional capacitive nonlinearity. Li et al. [38] also found that in MEMS DRG the main nonlinearity is caused by electrostatic and capacitive nonlinearities and proposed a method to reduce them. Since the main purpose of this paper is to deduce the relationship between nonlinearity and phase noise, the causation of nonlinearity is not discussed here. Nonlinear terms may cause a peak-frequency shift with amplitude variation, known as amplitude-stiffening (A-S) effect. He et al. [31] presented state-space approach based on LTV model and made a quantitative phase noise prediction of the automatic amplitude control (AAC) loop. Zhao et al. [32] proposed a system decomposition phase noise model that adopts a unified approach to deal with phase noise analysis in SOA. Ward and Duwel [28] adapted the LTV approach to provide more insight into the impact of nonlinear effects on phase noise with some success. Agrawal et al. [33,39] presented mathematical model that integrates the MEMS resonator and the oscillator circuitry nonlinearities and then analyzed the nonlinear effects on phase noise mathematically. 

Though these models have achieved better accuracy in different ways, they show less physical intuition, since most of them resolve the phase noise in a mathematical way. Moreover, little research has illustrated the influence of phase noise on gyroscope performance [40]. This paper proposes a phase noise model combined with the LTV model and A-S effect. Different from traditional analysis, this paper illustrates the LTV model in mechanical domain, which gains more physical insight into the origin of phase noise in MEMS DRG. Additionally, with the phase noise caused by A-S effect, the model established here suited well with the simulations. This paper also derives how the phase noise in drive loop affect the performance of MEMS DRG system. Some mechanical structural and circuit design guides are also proposed to reduce the impact of phase noise.

This paper organized as follows: the working principle of MEMS DRG is reviewed in Section 2. The mechanical domain time-varying phase noise model is established in Section 3. The influence of phase noise on the MEMS DRG system is described in Section 4. Some simulations and test results are shown in Section 5 and Section 6. The discussions and conclusions are given in Section 7 and Section 8.

## 2. Working Principle of the MEMS DRG

### 2.1. Two-Dimensional (2-D) Coriolis Vibratory Gyroscope Model

**Figure 1:**
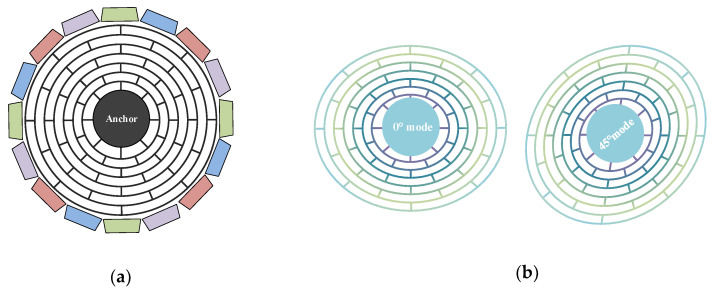
(**a**) Schematic view of the traditional disk resonator gyroscope. (**b**) Working modes of the disk resonator gyroscope (DRG).

MEMS DRG is a kind of solid-state wave gyroscope and its operational principle is based on Coriolis force. In MEMS DRG, the *n* = 2 wineglass mode is used as the working mode owing to its balanced pattern. The wineglass mode is driven into a planar bending resonance and Coriolis coupling transfers energy into a degenerate mode of the same shape, which is oriented 45° away from the drive mode axis, shown as Figure 1. 

The 2-D equation of movement (EoM) for the MEMS gyroscope (ignored the nonlinearities) can be written as [41,42]:

(1) $$\left[\begin{array}{cc} m_{x} & 0\\ 0 & m_{y} \end{array}\right]\left[\begin{array}{c} \ddot{x}\\ \ddot{y} \end{array}\right]+\left[\begin{array}{cc} D_{xx} & D_{xy}\\ D_{yx} & D_{yy} \end{array}\right]\left[\begin{array}{c} \dot{x}\\ \dot{y} \end{array}\right]+\left[\begin{array}{cc} k_{xx} & k_{xy}\\ k_{yx} & k_{yy} \end{array}\right]\left[\begin{array}{c} x\\ y \end{array}\right]=\left[\begin{array}{cc} 0 & 2nk\Omega_{z}(t)m_{y}\\ -2nk\Omega_{z}(t)m_{x} & 0 \end{array}\right]\left[\begin{array}{c} \dot{x}\\ \dot{y} \end{array}\right]+\left[\begin{array}{c} F_{x}\\ F_{y} \end{array}\right]$$

where:

(2) $$\begin{array}{l} \omega^{2}=\frac{\omega_{x}^{2}+\omega_{y}^{2}}{2};\frac{1}{\tau }=\frac{1}{2}\left(\frac{1}{\tau_{x}}+\frac{1}{\tau_{y}}\right);\omega \Delta \omega_{xy}=\frac{\omega_{x}^{2}-\omega_{y}^{2}}{2};\Delta \left(\frac{1}{\tau }\right)=\frac{1}{\tau_{x}}-\frac{1}{\tau_{y}}\\ \frac{D_{xx}}{m}=\frac{2}{\tau }+\Delta (\frac{1}{\tau })\cos2n\theta_{\tau };\frac{D_{xy}}{m}=\frac{D_{yx}}{m}=\Delta (\frac{1}{\tau })\sin2n\theta_{\tau };\frac{D_{yy}}{m}=\frac{2}{\tau }-\Delta (\frac{1}{\tau })\cos2n\theta_{\tau }\\ \frac{k_{xx}}{m}=\omega^{2}-\omega \Delta \omega_{xy}\cos2n\theta_{\omega };\frac{k_{yy}}{m}=\omega^{2}+\omega \Delta \omega_{xy}\cos2n\theta_{\omega };\frac{k_{xy}}{m}=\frac{k_{yx}}{m}=\omega \Delta \omega_{xy}\sin2n\theta_{\omega } \end{array}$$

In Equations (1) and (2), we assume *m_x_* = *m_y_* = *m*, *n* = 2 is the wineglass mode, *k* = 0.4 is the angular gain for DRG, *ω*_x_ and *ω*_y_ are the natural frequencies of the two modes, *τ*_x_ and *τ*_y_ are the principal damping time constants, *τ* is the decaying time constant, *ω* is resonance frequency, ∆(*1/τ*) refers to the damping asymmetry, ∆*ω_xy_* referred to frequency split, *θ_ω_* is the azimuth of the *ω*_y_ normal-mode axis measured from x direction, and *θ*_τ_ is the azimuth of the *τ*_x_ damping axis. 

In order to improve the performance of gyroscope, we need to minimum damping asymmetry and frequency split, which can be achieved by applying quadrature control loop in the DRG system. According to recent literature, the ∆*f_xy_* and ∆(*1/τ*) of DRG can be reduced to approximately 20 mHz and 70 μHz, respectively, after frequency tuning [43]. Thus, the mechanical coupling (cross-channel coupling) between the two axes of the DRG can be largely reduced [15]. 

### 2.2. The Working Principle of Drive Loop

**Figure 2:**
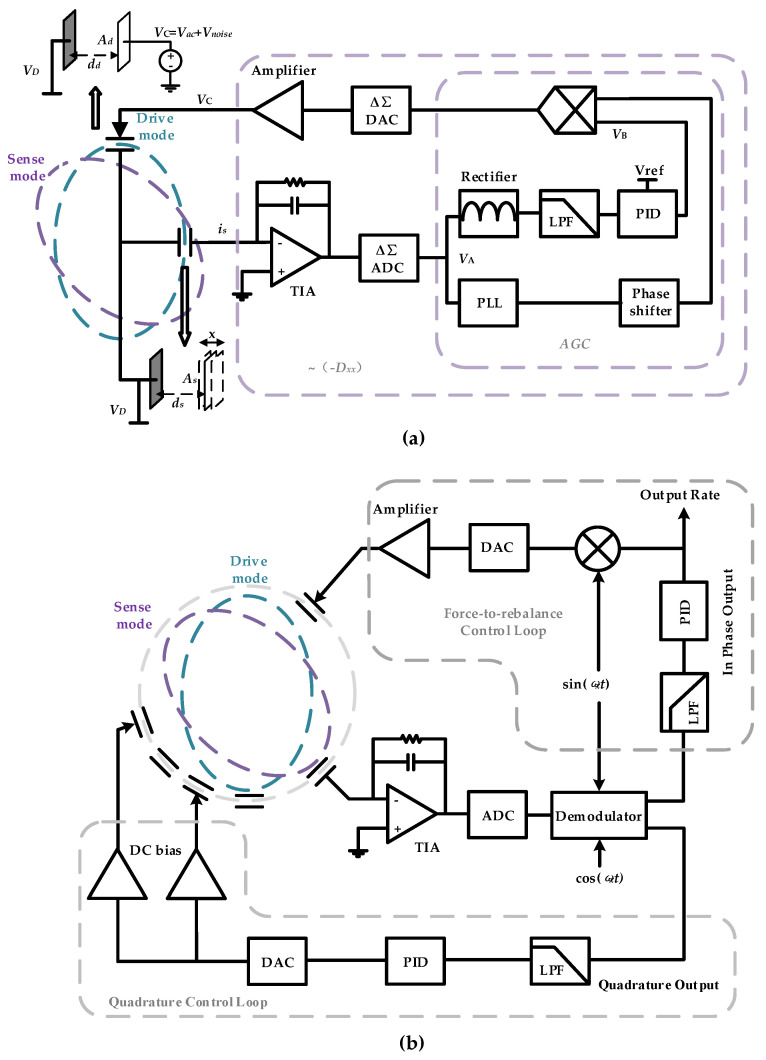
Overall scheme of DRG control circuit (**a**) Schematic diagram of self-excitation drive loop. (**b**) Schematic diagram of force-to-rebalance control loop and quadrature control loop.

The total DRG circuit combines with four loops—frequency control loop and amplitude control loop to maintain the frequency and amplitude stabilities of the drive mode, quadrature control loop to eliminate frequency split, and force-to-rebalance control loop to control the azimuth angle of resonator vibration. Although there are many different kinds of implementation methods of close loop, and each of them possess distinctive merit, the close loop principle is the same [44]. In this paper, a traditional digital control loop is chosen due to its easy to configure parameters. Figure 2 gives the schematic of the DRG control circuit.

Any practical oscillator has fluctuations in its amplitude and frequency. Thus, the output is more generally given by:

(3) $$x(t)=A(t)\cdot f[\omega_{x}t+\varphi_{pn}(t)]$$

where *φ_pn_*(*t*) and *A*(*t*) are the phase and amplitude fluctuations on time. In gyroscope system, the effect of amplitude fluctuation is reduced by amplitude limiting mechanism and can be practically eliminated by the AGC control circuit in drive loop, while the phase noise cannot be reduced in the same manner. Therefore, the *x*-directional (drive-directional) displacement can be written as:

(4) $$x(t)=A_{x}\cdot \sin(\omega_{x}t+\varphi_{pn}(t))$$

where *A_x_* is the maximum displacement of the drive axis.

In order to analysis the drive loop intuitively and deduce the maximum displacement of the mass *A_x_*, the working principle of the drive circuit is reviewed here. The ideal dynamical equation of the drive mode can be written as:

(5) $$m_{x}\frac{d^{2}x}{dt^{2}}+D_{xx}\frac{dx}{dt}+k_{xx}x=F_{x}$$

Differentiate both sides of the function and substitute *v_x_* = *dx/dt*, we get:

(6) $$m_{x}\frac{d^{2}v_{x}}{dt^{2}}+D_{xx}\frac{dv_{x}}{dt}+k_{xx}v_{x}=\frac{dF_{x}}{dt}$$

where *v_x_* is the velocity of the mass towards drive axis. Equation (6) shows that, for a resonator system, *D_xx_dv_x_/dt* needs to be equal to *dF_x_/dt*, which means in a steady drive loop, drive force may offsets velocity losses due to the damping.

The schematic of self-excitation drive-circuit on gyroscope is shown in Figure 2a. The detection capacitor is seen as a plane-parallel capacitor with one plate connected with fixed voltage source *V_D_* and the other connected with a trans-impedance amplifier. The capacitance of the plane-parallel capacitor is:

(7) $$C_{s}=\frac{A_{s}\varepsilon_{0}\varepsilon_{r}}{d_{s}+x}$$

where *d_s_* is the distance between the sensing capacitor plates, *A_s_* is the area of the sensing capacitor plates, *ε*_0_ is the permittivity of free space, and *ε_r_* is the relative dielectric permittivity. Thus, the quantity of electricity between the capacitor plates is:

(8) $$Q_{s}=C_{s}V_{D}=\frac{A_{s}\varepsilon_{0}\varepsilon_{r}}{d_{s}+x}V_{D}$$

and the output current is:

(9) $$i_{s}=\frac{dQ_{s}}{dt}=\frac{dC_{s}}{dt}V_{D}\approx -\frac{A_{s}\varepsilon_{0}\varepsilon_{r}}{d_{s}{}^{2}}V_{D}\frac{dx}{dt}=-\frac{A_{s}\varepsilon_{0}\varepsilon_{r}}{d_{s}{}^{2}}V_{D}v_{x}=k_{i/v}v_{x}$$

where we assume the fact *x* << *d_s_* and *k_i/v_* denotes the coefficient from velocity to output current. Equation (9) shows that the output current of the detection capacitor is proportional to the velocity of the mass. Thus, the drive loop can be viewed as an adjustable damping system. The system senses the velocity and adjusted its damping coefficient. When the drive loop works steady, the equivalent damping coefficient just equal to −*D_xx_*. The voltage before AGC is:

(10) $$V_{A}(t)=R_{V/i}\cdot i_{s}=k_{i/v}\cdot R_{V/i}\cdot v_{x,\max}\sin\omega_{x}t$$

where $v_{x,\max}$ is the maximum velocity of the mass towards drive axis and $R_{V/i}$ is the impedance between the input of TIA and AGC. The Rectifier and Low-Pass Filter (LPF) detect the mean abstract value of *V_A_*(*t*) and PI forces this value equal to *V_ref_*:

(11) $$V_{ref}=k_{i/v}R_{V/i}v_{x,\max}\frac{2\omega_{0x}}{\pi }\int_{0}^{\frac{\pi }{2\omega_{0x}}}\sin\omega_{x}tdt=\frac{2k_{i/v}R_{V/i}v_{x,\max}}{\pi }$$

Thus, the maximum velocity and displacement can be given by:

(12) $$v_{x,\max}=\frac{\pi V_{ref}}{2k_{i/v}R_{V/i}}$$

(13) $$A_{x}=\frac{\pi V_{ref}}{2k_{i/v}R_{V/i}\omega_{x}}$$

## 3. Phase Noise in Gyroscope System

### 3.1. Force Noise Act on the Mass 

In practice drive loop, the drive AC voltage together with noise voltage act on the drive capacitor, and both may produce force acting on the mass—AC voltage produces the desired force and noise voltage generates the unwanted. We call the unwanted force caused by noise voltage “force noise” here. The force act on the mass can be deduced as:

(14) $$F_{d}=-\frac{\partial E_{d}}{\partial (d_{d}+x)}=-\frac{\partial C_{d}}{\partial (d_{d}+x)}\frac{V_{d}{}^{2}}{2}=\frac{A_{d}\varepsilon_{0}\varepsilon_{r}V_{d}{}^{2}}{2{\left(d_{d}+x\right)}^{2}}\approx \frac{A_{d}\varepsilon_{0}\varepsilon_{r}V_{d}{}^{2}}{2d_{d}{}^{2}}$$

where *E_d_* is the energy storage in the diving capacitor, *d_d_* is the distance between the capacitor plates, *A_d_* is the area of the capacitor plates, and *V_d_* is the voltage on the capacitor. The approximation in Equation (14) assumes *x* << *d_s_*. However, the displacement *x* may change the electrical spring and cause nonlinear spring constants [39]. The drive voltage, shown in Figure 2a, can be written as:

(15) $$V_{d}(t)=V_{D}+V_{AC}\cos(\omega_{x}t)+V_{d,noise}(t)$$

where *V_AC_* is the amplitude of ac voltage and *V_d,noise_* (*t*) is the voltage noise on the drive capacitor. Thus, the force *F_d_* can be deduced as:

(16) $$\begin{array}{ll} F_{d} & =\frac{A_{d}\varepsilon_{0}\varepsilon_{r}V_{d}^{2}(t)}{2d_{d}{}^{2}}=\frac{A\varepsilon_{0}\varepsilon_{r}{\left(V_{D}+V_{AC}\cos\omega_{x}t+V_{d,noise}(t)\right)}^{2}}{2d_{d}{}^{2}}\\ & \approx \frac{A_{d}\varepsilon_{0}\varepsilon_{r}}{2d_{d}{}^{2}}V_{D}^{2}+\frac{A_{d}\varepsilon_{0}\varepsilon_{r}}{2d_{d}{}^{2}}V_{D}V_{AC}\cos\omega_{x}t+\frac{A_{d}\varepsilon_{0}\varepsilon_{r}}{2d_{d}{}^{2}}V_{D}V_{d,noise}(t)\\ & =F_{D}+F_{AC}\cos\omega_{x}t+F_{d,noise}(t) \end{array}$$

where *F_D_* is useless for the oscillation since it is a fixed force, *F_AC_*cos*ω_x_t* forces the mass vibrate at resonance frequency, and the unwanted force noise is:

(17) $$F_{d,noise}(t)=\frac{A_{d}\varepsilon_{0}\varepsilon_{r}}{2d_{d}{}^{2}}V_{D}V_{d,noise}(t)=k_{F/V}V_{d,noise}(t)$$

where *k_F/V_* denotes the coefficient from noise voltage to the force noise act on the mass. 

### 3.2. The Established Phase Noise Model in Gyroscope System 

#### 3.2.1. Phase Noise Caused by Injected Force Noise 

**Figure 3:**
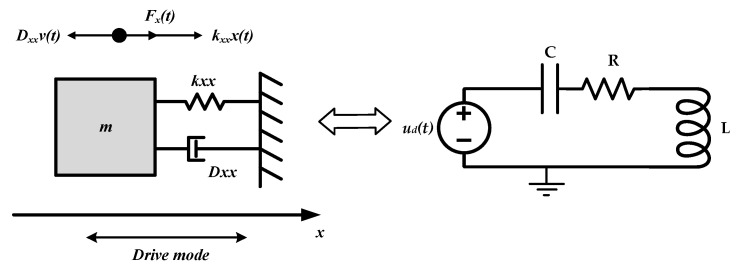
Comparison between the mechanical and electrical model.

The established phase noise model is analyzed in mechanical domain. By transferring mechanical domain into electric domain, gyroscope oscillation is a series resonant rather than parallel resonant, which is different from Hajimiri’s analysis [22]. In order to analyze the proposed model intuitively, we compare the mechanical resonance system with the series LC circuit in Figure 3. The comparison of parameters is listed in Table 1.

When the drive loop works steadily, the velocity and the displacement of the mass can be expressed as:

(18) $$v(t)=v_{x,\max}\cdot \cos\omega_{x}t$$

(19) $$x(t)=v_{x,\max}\cdot \sqrt{\frac{m_{x}}{k_{xx}}}\sin\omega_{x}t$$

Suppose that an extra force *F_inj_* acts on the mass, the displacement and velocity cannot response instantly due to the inertia effect, but the accelerated velocity is changed by *F_inj_/m_x_* (just as when an extra voltage injected in the series LC circuit, the current in the loop and the voltage on capacitance cannot response quickly but the voltage on the inductance can). Then consider that the external force lasts only a little while, Δ*t* (Δ*t*→0); in other words, suppose an injected impulse Δ*J = F_inj_*Δ*t* acts on the mass, the velocity of the mass will be changed by Δ*J/m_x_* instantly. However, the displacement cannot reflect fast enough due to the extreme short time (just as when a flux linkage Δ*ψ = u_inj_*Δ*t* acts on the series LC circuit, the current is changed by Δ*ψ/L*, but the voltage on the capacitor cannot reflect fast enough). Thus, the amplitude and phase fluctuation can be expressed as:

(20) $$(v_{x,\max}+\Delta v)\cdot \cos(\omega_{x}t+\Delta \varphi )=v_{x,\max}\cos\omega_{x}t+\frac{\Delta J}{m_{x}}$$

(21) $$(v_{x,\max}+\Delta v)\cdot \sqrt{\frac{m_{x}}{k_{xx}}}\sin(\omega_{x}t+\Delta \varphi )=v_{x,\max}\sqrt{\frac{m_{x}}{k_{xx}}}\sin\omega_{x}t$$

where ∆*v* and ∆*φ* present amplitude and phase fluctuation caused by the injected impulse. In a real system, where the fluctuation due to impulse is small enough, we assume that cos∆*φ* ≈ 1, sin∆*φ* ≈ ∆*φ*, by expanding the trigonometric function; thus Equations (20) and (21) can be rewritten as:

(22) $$(v_{x,\max}+\Delta v)(\cos\omega_{x}t-\Delta \varphi \sin\omega_{x}t)=v_{x,\max}\cos\omega_{x}t+\frac{\Delta J}{m}$$

(23) $$(v_{x,\max}+\Delta v)\cdot \sqrt{\frac{m_{x}}{k_{xx}}}(\sin\omega_{x}t+\Delta \varphi \cos\omega_{x}t)=v_{x,\max}\sqrt{\frac{m_{x}}{k_{xx}}}\sin\omega_{x}t$$

By solving Equations (22) and (23) simultaneously, the amplitude and phase fluctuation can be deduced as:

(24) $$\Delta v=\frac{\Delta J}{m_{x}}\cos\omega_{x}t$$

(25) $$\Delta \varphi =-\frac{\Delta J}{m_{x}v_{x,\max}}\sin\omega_{x}t$$

**Figure 4:**
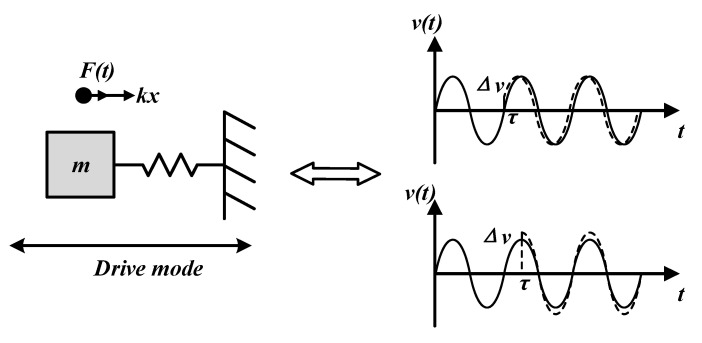
Impulse response of mechanical oscillation.

Equations (24) and (25) show that the impulse response for mechanical oscillation depends on the time when the impulse is injected. For example, when the impulse injects at the peak of velocity, the amplitude of the velocity will change significantly but the phase will change little. When the impulse is injected at time *v*(*t*) = 0, the phase may change obviously but the amplitude may change slightly, as Figure 4 shows.

For a more generally case, the unit impulse response for excess amplitude and phase can be expressed as:

(26) $$h_{v}(t,\tau )=\frac{\Delta v(t,\tau )}{\Delta J}=\frac{H(\omega_{x}\tau )}{m_{x}}u(t-\tau )$$

(27) $$h_{\varphi }(t,\tau )=-\frac{\Delta \varphi (t,\tau )}{\Delta J}=-\frac{\Gamma (\omega_{x}\tau )}{m_{x}v_{x}}u(t-\tau )$$

where H(*x*) can be viewed as the impulse-amplitude-sensitive function and Γ(*x*) can be viewed as the impulse-phase-sensitive function. They are dimensionless, frequency- and amplitude-independent periodic functions that describe how much amplitude shift and phase shift result from applying a unit impulse. For an ideal sine oscillation, H(*ω_x_τ*) = cos(*ω_x_τ*) and Γ(*ω_x_τ*) = sin(*ω_x_τ*); thus, Equations (26) and (27) are equivalent to Equations (24) and (25), respectively. More generally, in a practical gyroscope system, they can be expanded in a Fourier series:

(28) $$H(\omega_{x}\tau )=\frac{c_{v,0}}{2}+\sum_{n=1}^{\infty }c_{v,n}\cos n\omega_{x}\tau$$

(29) $$\Gamma (\omega_{x}\tau )=\frac{c_{\varphi ,0}}{2}+\sum_{n=1}^{\infty }c_{\varphi ,n}\sin n\omega_{x}\tau$$

where the coefficients *c_v,n_* and *c_φ,n_* are real-valued coefficients of the *n*th harmonic of H(*x*) and Γ(*x*), respectively.

According to Equation (27), the impulse-phase-sensitive function is practically linear and the impulse response for the system is a step whose amplitude depends periodically on the time *τ* when the impulse is injected. Therefore, using the superposition integral, the excess phase $\varphi_{LTV}(t)$ predicted by LTV model can be calculated as:

(30) $$\begin{array}{ll} \varphi_{LTV}(t) & =\int_{-\infty }^{\infty }h_{\varphi }(t,\tau )F_{noise}(\tau )d\tau =-\frac{1}{m_{x}v_{x}}\int_{-\infty }^{t}\Gamma (\omega_{x}\tau )F_{noise}(\tau )d\tau \\ & =-\frac{1}{m_{x}v_{x}}\left[\frac{c_{\varphi ,0}}{2}\int_{-\infty }^{t}F_{noise}(\tau )d\tau +\sum_{n=1}^{\infty }c_{\varphi ,n}\int_{-\infty }^{t}F_{noise}(\tau )\sin(n\omega_{x}\tau )d\tau \right] \end{array}$$

Since in a practical circuit the amplitude of velocity noise varies with the frequency, we analyzed the excess phase by injecting different frequency sinusoidal perturbation force:

(31) $$F_{inject}(t)=F_{inject}\sin(n\omega_{x}\pm \Delta \omega )t$$

Considering that a low frequency sinusoidal perturbation force act on the mass, which is given by *F_inject_,*_0_(*t*) = *F_inject_,*_0_ sin(∆*ωt*) at a frequency of ∆*ω* << *ω_x_*, the phase fluctuation is:

(32) $$\varphi_{LTV,0}(t)=-\frac{c_{\varphi ,0}F_{inject,0}}{2m_{x}v_{x}}\int_{-\infty }^{t}\sin(\Delta \omega \tau )d\tau =\frac{c_{\varphi ,0}F_{inject,0}(t)\cos(\Delta \omega t)}{2m_{x}v_{x}\Delta \omega }$$

where $\varphi_{LTV,0}(t)$ is the excess phase caused by the low frequency perturbation force and *F_inject_*_,0_ is the amplitude of perturbation force at the frequency of ∆*ω*.

Considering a sinusoidal perturbation force close to the carrier injected into the resonance system, given by *F_inject_*_,1_(*t*) = *F_inject_*_,1_ sin[(*ω_x_* ± ∆*ω*)*t*] at a frequency of ∆*ω* << *ω_x_*, the phase fluctuation is:

(33) $$\varphi_{LTV,1}(t)=\frac{c_{\varphi ,1}F_{inject,1}(t)\cos(\Delta \omega t)}{2m_{x}v_{x}\Delta \omega }$$

**Figure 5:**
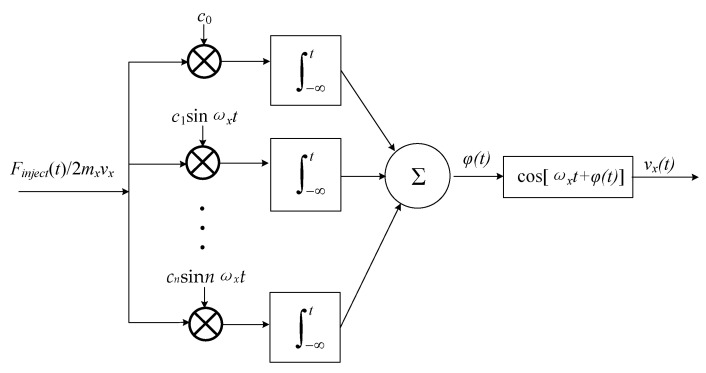
Linear time-variant (LTV) model of the self-excited loop of the micro-gyro.

More generally, when applying a perturbation force close to any integer multiple of the oscillation frequency, the phase fluctuation will result in a general phase change given by:

(34) $$\varphi_{LTV,n}(t)=\frac{c_{\varphi ,n}F_{inject,n}(t)\cos(\Delta \omega t)}{2m_{x}v_{x}\Delta \omega }$$

where $\varphi_{LTV,n}(t)$ is the LTV excess phase caused by frequency perturbation force near *n* multiple of the oscillation frequency, *F_inject_*_,n_ is the amplitude of force noise at the frequency of *nω_x_ ±* ∆*ω*. The corresponding sequence of mathematical operations is shown graphically in Figure 5.

Thus, the total phase fluctuation predicted by LTV model $\varphi_{LTV}(t)$ is given by:

(35) $$\varphi_{LTV}(t)=\frac{\cos(\Delta \omega t)}{2m_{x}v_{x}\Delta \omega }\sum_{n=0}^{\infty }c_{\varphi ,n}F_{inject,n}$$

Substituting $\varphi_{LTV}(t)$ from Equation (35) into Equation (4) results in a single-tone phase modulation for the *x*-directional displacement of the mass:

(36) $$\begin{array}{ll} x(t) & =A_{x}\cdot \sin(\omega_{x}t+\varphi_{LTV}(t))=A_{x}[\sin\omega_{x}t+\varphi_{LTV}(t)\cos\omega_{x}t]\\ & =A_{x}[\sin\omega_{x}t+\frac{\sum_{n=0}^{\infty }c_{\varphi ,n}F_{inject,n}}{2m_{x}v_{x}\Delta \omega }\cos(\Delta \omega t)\cos\omega_{x}t]\\ & =A_{x}\sin\omega_{x}t+\frac{A_{x}\sum_{n=0}^{\infty }c_{\varphi ,n}F_{inject,n}}{4m_{x}v_{x}\Delta \omega }[\cos(\omega_{x}+\Delta \omega )t+\cos(\omega_{x}-\Delta \omega )t] \end{array}$$

Therefore, the sideband power relatives to the carrier caused by injected force is given by:

(37) $$P_{SBC}(\Delta \omega )=10\cdot \log{\left(\frac{\sum_{n=0}^{\infty }c_{\varphi ,n}F_{inject,n}}{4m_{x}v_{x}\Delta \omega }\right)}^{2}$$

Now, consider the power spectral density of the voltage noise on the capacitor is ${\bar{V}}_{d,noise}^{2}/\Delta f$, according to Equation (17), the force noise with a power spectral density is $k_{F/V}^{2}{\bar{V}}_{d,noise}^{2}/\Delta f$. Note that *F_inject,n_* in Equation (35) represents the peak amplitude; hence, $F_{inject,n}^{2}/2=k_{F/V}^{2}{\bar{V}}_{d,noise}^{2}/\Delta f$ for Δ*f =* 1 Hz. Then, the total single sideband phase noise spectral density caused by injected force in dB below the carrier per unit bandwidth is given by:

(38) $$L(\Delta \omega )=10\cdot \log\left(\frac{k_{F/V}^{2}\sum_{n=0}^{\infty }c_{\varphi ,n}^{2}{\bar{V}}_{d,noise}^{2}/\Delta f}{8m_{x}^{2}v_{x}^{2}\Delta \omega^{2}}\right)=10\cdot \log\left(\frac{k_{F/V}^{2}\sum_{n=0}^{\infty }c_{\varphi ,n}^{2}{\bar{V}}_{d,noise}^{2}/\Delta f}{8m_{x}k_{xx}A_{x}^{2}\Delta \omega^{2}}\right)$$

According to Parseval’s relation theorem:

(39) $$\sum_{n=0}^{\infty }c_{\varphi ,n}^{2}=\frac{1}{\pi }{\int_{0}^{2\pi }\left|\Gamma (x)\right|}^{2}dx=2\Gamma_{\mathrm{rms}}^{2}$$

where Γ_rms_ is the rms value of Γ(x). The phase noise spectrum caused by white noise can be deduced as:

(40) $$L_{LTV,1/f^{2}}(\Delta \omega )=10\cdot \log\left(\frac{\Gamma_{rms}^{2}k_{F/V}^{2}{\bar{V}}_{d,noise}^{2}/\Delta f}{4m_{x}k_{xx}A_{x}^{2}\Delta \omega^{2}}\right)$$

Equation (40) describes the 1/*f*^2^ region phase noise spectrum of gyroscope system caused by the force noise injection, since in CMOS technology, flicker noise dominated the low frequency noise spectrum. The 1/*f* voltage noise spectrum act on the capacitor can be described by:

(41) $${\bar{V}}_{d,noise,1/f}^{2}={\bar{V}}_{d,noise}^{2}\cdot \frac{\omega_{d,1/f}}{\Delta \omega }\;(\Delta \omega <\omega_{d,1/f})$$

where $\omega_{d,1/f}$ is the corner frequency of 1/*f* noise. The 1/*f*^3^ region phase noise spectrum predicted by LTV model can be deduced as:

(42) $$L_{LTV,1/f^{3}}(\Delta \omega )=10\cdot \log\left(\frac{c_{\varphi ,0}^{2}k_{F/V}^{2}{\bar{V}}_{C,noise}^{2}\omega_{d,1/f}/\Delta f}{8m_{x}k_{xx}A_{x}^{2}\Delta \omega^{3}}\right)$$

#### 3.2.2. Phase Noise Caused by A-S Effect

In practical MEMS oscillators, mechanical nonlinearities and electrical nonlinearities may cause first- and second-spring terms *k*_1_ and *k*_2_ [39]. The motion equation for the drive mode can be written as:

(43) $$m_{x}\frac{d^{2}x}{dt^{2}}+D_{xx}\frac{dx}{dt}+k_{xx}x+k_{1}x^{2}+k_{2}x^{3}=F_{x}$$

The nonlinear terms cause a peak-frequency shift with vibration-amplitude increases. The real time frequency can be deduced as [45]:

(44) $$\begin{array}{ll} \omega (t) & =\omega_{x}\left[1+\left(\frac{3k_{2}}{8k_{xx}}-\frac{5k_{1}^{2}}{12k_{xx}^{2}}\right)A^{2}(t)\right]=\omega_{x}\left[1+\lambda {\left(A_{x}+\Delta A(t)\right)}^{2}\right]\\ & \approx \omega_{x}\left(1+\lambda A_{x}^{2}\right)\left(1+\frac{2\lambda A_{x}}{1+\lambda A_{x}^{2}}\Delta A(t)\right)\\ & ={\omega^{\prime }}_{x}\left(1+\frac{2\lambda A_{x}}{1+\lambda A_{x}^{2}}\Delta A(t)\right) \end{array}$$

where ${\omega^{\prime }}_{x}$ is the resonance frequency at $A_{x}$, ΔA(t) is amplitude fluctuation. The addition phase cause by A-S effect is:

(45) $$\varphi_{A-S}(t)={\omega^{\prime }}_{x}\int_{0}^{t}\frac{2\lambda A_{x}}{1+\lambda A_{x}^{2}}\Delta A(t)dt=\omega_{x}\int_{0}^{t}2\lambda A_{x}\Delta A(t)dt$$

Since an explicit automatic gain control (AGC) is employed, the amplitude fluctuation caused by force injection will finally be eliminated. Thus, we cannot use the superposition integral of the impulse-amplitude-sensitive function to predict the amplitude change, which is different from the equation deduced from $\varphi_{pn,s}(t)$. However, the integral term (I) in the AGC forces the vibration amplitude proportional to the reference voltage in low frequency. Based on this principal, the fluctuation on *V_ref_* directly influences ΔA(t):

(46) $$\Delta A(t)=\frac{\Delta V_{ref}(t)}{V_{ref}}A_{x}$$

Considering a sinusoidal voltage injected in the voltage reference:

(47) $$\Delta V_{ref}(t)=V_{ref,inj}\sin(\Delta \omega t)$$

Combining Equations (13), (45) and (46) with Equation (47), we can deduce the excess phase caused by the A-S effect:

(48) $$\varphi_{A-S}(t)=\frac{-\pi \lambda A_{x}V_{ref,inj}}{k_{i/v}R_{V/i}\Delta \omega }\cos(\Delta \omega t)$$

Similar to Equation (37), the sideband power at *ω_x_* + Δ*ω* relative to the carrier caused by A-S effect given by:

(49) $$P_{SBC}(\Delta \omega )=10\cdot \log{\left(\frac{\lambda \pi A_{x}V_{ref,inj}}{2k_{i/v}R_{V/i}\Delta \omega }\right)}^{2}$$

Suppose the 1/*f* voltage noise spectrum added on the reference voltage is:

(50) $${\bar{V}}_{ref,noise,1/f}^{2}={\bar{V}}_{ref,noise}^{2}\cdot \frac{\omega_{ref,1/f}}{\Delta \omega }\;(\Delta \omega <\omega_{ref,1/f})$$

where $\omega_{ref,1/f}$ is the corner frequency of 1/*f* noise on reference voltage. The 1/*f*^3^ region phase noise spectrum caused by the A-S effect is:

(51) $$L_{A-S}(\Delta \omega )=10\cdot \log\left(\frac{\lambda^{2}\pi^{2}A_{x}^{2}V_{ref,noise}^{2}\omega_{ref,1/f}/\Delta f}{4k_{i/v}^{2}R_{V/i}^{2}\Delta \omega^{3}}\right)$$

#### 3.2.3. Total Phase Noise 

The total excess phase is the sum of excess phase of LTV model and A-S effect. Noted that the phase of injected voltage is arbitrary, the total phase fluctuation is simply written by:

(52) $$\varphi_{pn}(t)=\alpha \sin(\Delta \omega t+\varphi_{eq})$$

where α and $\varphi_{eq}$ are the equivalent amplitude and phase caused by different noise injection. According to Equations (40), (42), and (51), the total phase noise spectrum is given by:

(53) $$L(\Delta \omega )=10\log\left(\frac{\Gamma_{rms}^{2}k_{F/V}^{2}{\bar{V}}_{C,noise}^{2}/\Delta f}{4m_{x}k_{xx}A_{x}^{2}\Delta \omega^{2}}+\frac{c_{0}^{2}k_{F/V}^{2}{\bar{V}}_{C,noise}^{2}\omega_{C,1/f}/\Delta f}{8m_{x}k_{xx}A_{x}^{2}\Delta \omega^{3}}+\frac{\lambda^{2}\pi^{2}A_{x}^{2}V_{ref,noise}^{2}\omega_{ref,1/f}/\Delta f}{4k_{i/v}^{2}R_{V/i}^{2}\Delta \omega^{3}}\right)$$

Equation (53) evaluates the total phase noise in drive loop. In order to reduce the phase noise, we need to optimize the design both of the mechanical structural and diving circuit. Regarding the mechanical structural aspect, increasing the mass and stiffness benefits the reduction of the impact of the force noise injection; reducing the nonlinearity of the spring can lower the 1/*f*^3^ phase noise. From a circuit design aspect, we need to decrease the voltage noise acting on the drive capacitor near the fundamental frequency and reduce the low frequency noise of the voltage reference. The displacement of the drive axis is controlled by a voltage reference. A larger displacement may reduce the impact of the force noise injection, but deteriorate the 1/*f*^3^ phase noise caused by the A-S effect. Thus, attention should be paid to design the proper displacement amplitude.

## 4. The Influence of Time-Varying Phase Noise on the MEMS DRG System 

### 4.1. y-Directional Displacement

According to the 2-D coriolis vibratory gyroscope model, the movement function of the *y*-directional resonator can be written as:

(54) $$m_{y}\ddot{y}+D_{yy}\dot{y}+k_{yy}y=-(D_{yx}+2nk\Omega m_{x})\dot{x}-k_{yx}x+F_{y}$$

where *k_yx_, D_yx_,* and *F_y_* will cause the errors of the mechanical quadrature signal, nonproportional damping, and direct excitation of secondary resonator, respectively [46]. The displacement of *x*-axis can be written as:

(55) $$x(t)=A_{x}\cdot \sin(\omega_{x}t+\varphi_{pn}(t))=A_{x}\sin(\omega_{x}t+\alpha \sin(\Delta \omega t+\varphi_{eq}))$$

Assuming ∆*ω*<<*ω_x_*, cos∆*φ* ≈ 1, sin∆*φ* ≈ ∆*φ*, we get:

(56) $$x(t)\approx A_{x}\sin\omega_{x}t-\frac{\alpha A_{x}}{2}[\sin(\omega_{x}t-\Delta \omega t-\varphi_{eq})-\sin(\omega_{x}t+\Delta \omega t+\varphi_{eq})]$$

(57) $$\dot{x}(t)\approx A_{x}\omega_{x}\cos\omega_{x}t-\frac{\alpha A_{x}\omega_{x}}{2}[\cos(\omega_{x}t-\Delta \omega t-\varphi_{eq})-\cos(\omega_{x}t+\Delta \omega t+\varphi_{eq})]$$

The coupled force caused by the primary resonator excitation signal can be written as:

(58) $$F_{y}=\beta F_{AC}\cos\omega_{x}t$$

where *β* is the magnitude of direct excitation. The transfer function of the secondary resonator from force to displacement can be written as:

(59) $$H_{y/F}(j\omega )=\frac{Q_{y}}{k_{yy}(1+j2Q_{y}\frac{\omega -\omega_{y}}{\omega_{y}})}$$

where *Q_y_* is the quality factor of the secondary resonator. For high *Q* system, the magnitude of the transfer function *G_y/F_*(*jω*) = |*H_y/F_* (*jω*)| decreases fast deviating from the resonance frequency. Thus, assuming ∆*ω_xy_* < ∆*ω* << *ω_y_*, we get *G_y/F_*(*j*(*ω_y_* ± ∆*ω*)) << *G_y/F_* (*jω_x_*), which reflects in match-mode MEMS DRG the phase noise in *y*(*t*) can be largely reduced by the frequency-selection characteristic of the transfer function, and can thus be neglected. By deducing Equation (54), we get:

(60) $$\begin{array}{ll} y(t)= & -G_{y/x}(j\omega_{x})A_{x}\omega_{x}2nk\Omega m_{x}\cos(\omega_{x}t+\phi_{yx})\\ & -G_{y/x}(j\omega_{x})A_{x}\omega_{x}D_{yx}\cos(\omega_{x}t+\phi_{yx})\\ & -G_{y/x}(j\omega_{x})\beta F_{AC}\cos(\omega_{x}t+\phi_{yx})\\ & +G_{y/x}(j\omega_{x})k_{yx}A_{x}\sin(\omega_{x}t+\phi_{yx}) \end{array}$$

(61) $$\phi_{yx}=\arctan\frac{\omega_{y}}{2\Delta \omega_{xy}Q_{y}}$$

### 4.2. Synchronous Demodulation

Assuming the gain of the secondary resonator detection circuit (displacement-to-voltage converter) is *G_V/y_*, the output voltage before demodulator can be given by:

(62) $$\begin{array}{ll} V_{\sec,dem,in}(t)= & -G_{y/x}(j\omega_{x})G_{V/y}A_{x}\omega_{x}2nk\Omega m_{x}\cos(\omega_{x}t+\phi_{yx})\\ & -G_{y/x}(j\omega_{x})G_{V/y}A_{x}\omega_{x}D_{yx}\cos(\omega_{x}t+\phi_{yx})\\ & -G_{y/x}(j\omega_{x})G_{V/y}\beta F_{AC}\cos(\omega_{x}t+\phi_{yx})\\ & +G_{y/x}(j\omega_{x})G_{V/y}k_{yx}A_{x}\sin(\omega_{x}t+\phi_{yx}) \end{array}$$

The modulation signal is chosen from the drive loop and can be written as:

(63) $$V_{drive,dem}(t)=A_{drive,dem}\cos(\omega_{x}t+\varphi_{pn}(t))$$

where *A_drive,dem_* is the amplitude of the modulation signal. Therefore, the resulting output signals from the demodulator (after low frequency filter) is:

(64) $$\begin{array}{ll} S_{sem,dem,out}(t)= & -G_{y/x}(j\omega_{x})G_{V/y}A_{drive,dem}A_{x}\omega_{x}nk\Omega m_{x}[\cos\phi_{yx}+\varphi_{pn}(t)\sin\phi_{yx}]\\ & -G_{y/x}(j\omega_{x})G_{V/y}A_{drive,dem}A_{x}\omega_{x}D_{yx}[\cos\phi_{yx}+\varphi_{pn}(t)\sin\phi_{yx}]/2\\ & -G_{y/x}(j\omega_{x})G_{V/y}A_{drive,dem}\beta F_{AC}[\cos\phi_{yx}+\varphi_{pn}(t)\sin\phi_{yx}]/2\\ & +G_{y/x}(j\omega_{x})G_{V/y}A_{drive,dem}k_{yx}A_{x}[\sin\phi_{yx}-\varphi_{pn}(t)\cos\phi_{yx}]/2 \end{array}$$

To gain more insight into the result, Equation (64) needs to be reduced to input angular velocity. This can be done by dividing $-G_{V/y}G_{y/x}(j\omega_{x})A_{drive,dem}A_{x}\omega_{x}nkm_{x}\cos\phi_{yx}$ on both sides of the equation, leading to:

(65) $$\Omega_{dem,out}=\left(\Omega +\frac{D_{yx}}{2nkm_{x}}+\frac{\beta F_{AC}}{2A_{x}\omega_{x}nkm_{x}}\right)\left(1+\varphi_{pn}(t)\tan\phi_{yx}\right)-\frac{k_{yx}}{2nkm_{x}\omega_{x}}\left(\tan\phi_{yx}-\varphi_{pn}(t)\right)$$

Thus, the zero-rate output is:

(66) $$\Omega_{dem,out}=\frac{D_{yx}}{2nkm_{x}}+\frac{\beta F_{AC}}{2A_{x}\omega_{x}nkm_{x}}-\frac{k_{yx}}{2\omega_{x}nkm_{x}}\tan\phi_{yx}$$

The output error relating to the phase fluctuation is:

(67) $$\Omega_{pn}=\left[\left(\Omega +\frac{D_{yx}}{2nkm_{x}}+\frac{\beta F_{AC}}{2A_{x}\omega_{x}nkm_{x}}\right)\tan\phi_{yx}+\frac{k_{yx}}{2\omega_{x}nkm_{x}}\right]\varphi_{pn}(t)$$

Suppose the full scale of the input angular velocity is Ω*_FSR_*, the power spectrum density caused by the phase noise can be deduced as:

(68) $$L_{dem}(\Delta \omega )=20\log\left\{\left[\left(2\Omega +\frac{D_{yx}}{nkm_{x}}+\frac{\beta F_{AC}}{A_{x}\omega_{x}nkm_{x}}\right)\tan\phi_{yx}+\frac{k_{yx}}{2\omega_{x}nkm_{x}}\right]/\Omega_{FSR}\right\}+L(\Delta \omega )$$

This equation shows how the phase noise transfers into the low frequency noise in the output of the gyroscope system and affects the bias stability of the gyroscope. Apparently, to reduce the impact of the phase noise, we need to diminish the non-ideal factors in the mechanical structural aspect. From a circuit design aspect, we can use accurate phase adjustment technology between the modulation signal and demodulated signal to diminish $\tan\phi_{yx}$ item [47,48]. However, the structure parameters are changed with environmental factors and $\tan\phi_{yx}$ may change dramatically. Therefore, a strict quadrature control loop and phase adjustment circuit is needed for a high performance DRG system.

## 5. Numerical Simulations

**Figure 6:**
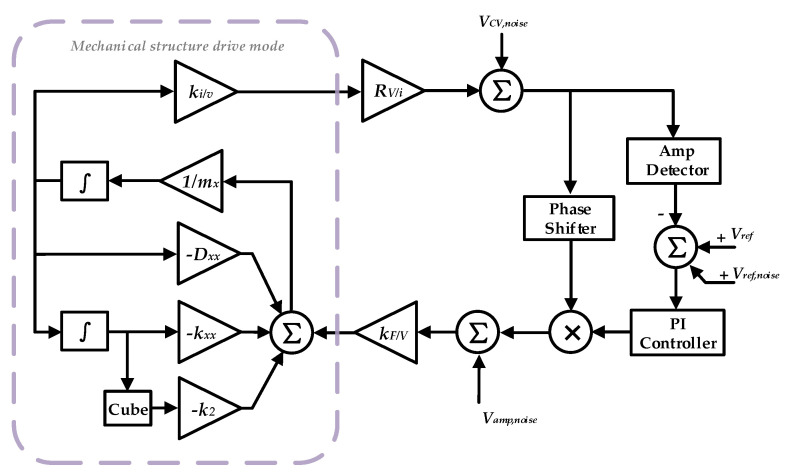
Time domain model for drive loop of DRG.

To verify the proposed phase noise model, a time domain model for the drive loop of DRG is established. The block diagram of the simulation model is shown in Figure 6 and the parameters for the simulation are listed in Table 2. In Figure 6, *V_CV,noise_*, *V_ref,noise_*, and *V_amp,noise_* represent the voltage noise caused by the charge amplifier, voltage reference, and drive amplifier, respectively. Since in steady working mode, the output of PI is near a DC voltage, the noise transfer function from the output of the charge amplifier to the drive node is just a scale coefficient. Therefore, *V_CV,noise_* and *V_amp,noise_* cause phase noise by generating force noise, and *V_ref,noise_* causes phase noise through the A-S effect.

**Figure 7:**
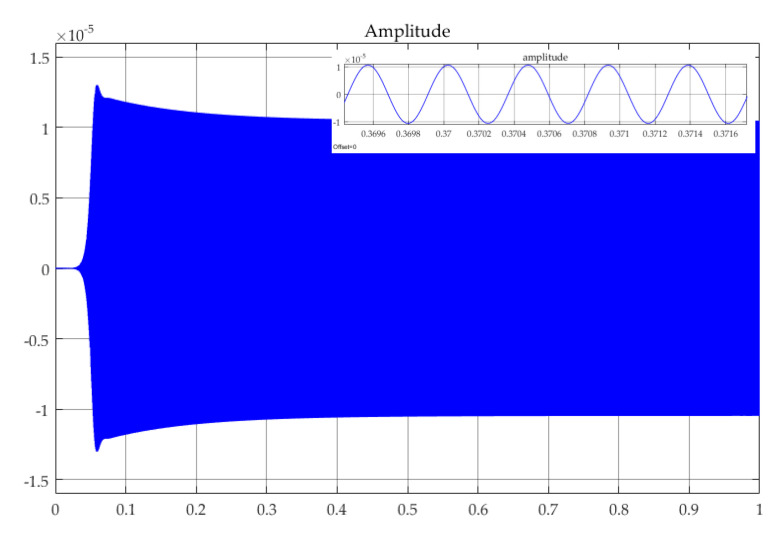
Transient waveform of the x-axis displacement.

Firstly, the phase fluctuation caused by the force noise injection is simulated. We set *k*_2_ = 0 to avoid the influence of A-S effect. According to Equations (2) and (13), the resonance frequency and maximum amplitude are *f_x_* = 2.199 kHz and *A_x_* = 10.47 μm, respectively, which are in accordance with the simulated transient waveform shown in Figure 7.

**Figure 8:**
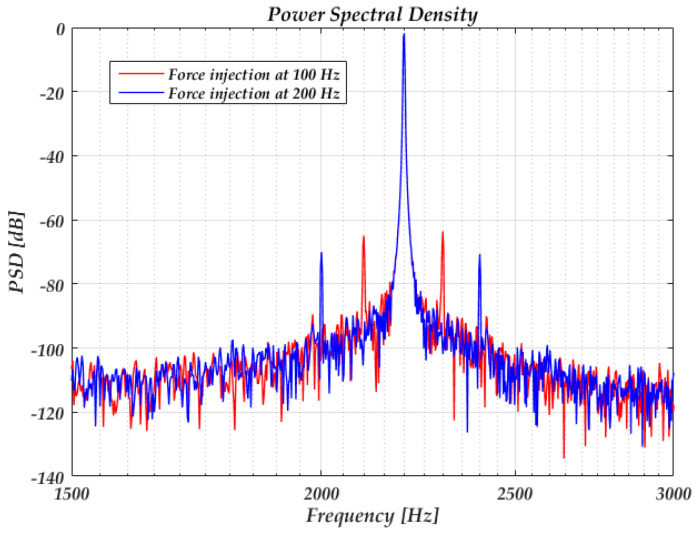
Simulated power spectrum of the output with force injection.

Since $c_{\varphi ,0}$ in Equation (42) is the DC value of the ISF and is dependent on the rise- and fall-time symmetry of the sine wave, the 1/*f*^3^ noise caused by the force noise through $c_{\varphi ,0}$ can be hardly simulated due to the high-quality factor (160 k according to the model). Figure 8 shows the power spectrum of the C-V output with a force injection at ∆*ω* = 100 Hz and 200 Hz, respectively, related to the resonance frequency. The peak injecting voltage is 10 mV. According to Equation (37), the predicted sideband powers are −65 dB and −71 dB, respectively, which coincide well with the simulated results.

**Figure 9:**
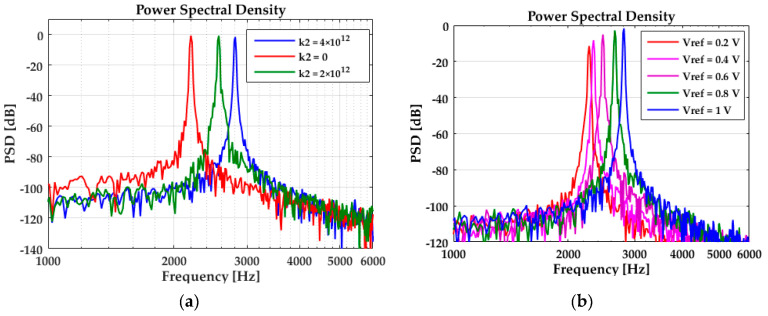
(**a**) Resonance frequency varies with nonlinear coefficient; (**b**) resonance frequency varies with amplitude.

Phase fluctuation caused by A-S effect is simulated next. Figure 9 shows the simulated resonance frequency varies with nonlinear coefficient (*k*_2_) and peak amplitude. In Figure 9a, the reference voltage *V*_ref_ = 1 V, *k*_2_ is set to 0, 2 × 10^12^, 4 × 10^12^, respectively. The simulated results are 2197 Hz, 2564 Hz, and 2808 Hz, which suit well with the predicted results of 2199 Hz, 2595 Hz, and 2990 Hz according to Equation (44). Figure 9b simulated the resonance frequency, which varies with the amplitude. In this simulation, *k*_2_ = 4 × 10^12^, the reference voltage *V*_ref_ is set to 0.2 V, 0.4 V, 0.6 V, 0.8 V, and 1 V, respectively. The simulated results of resonance frequency are 2274 Hz, 2335 Hz, 2472 Hz, 2655 Hz, and 2808 Hz, which are well-suited with the predicted results of 2232 Hz, 2327 Hz, 2486 Hz, 2708 Hz, and 2990 Hz according to Equation (44).

**Figure 10:**
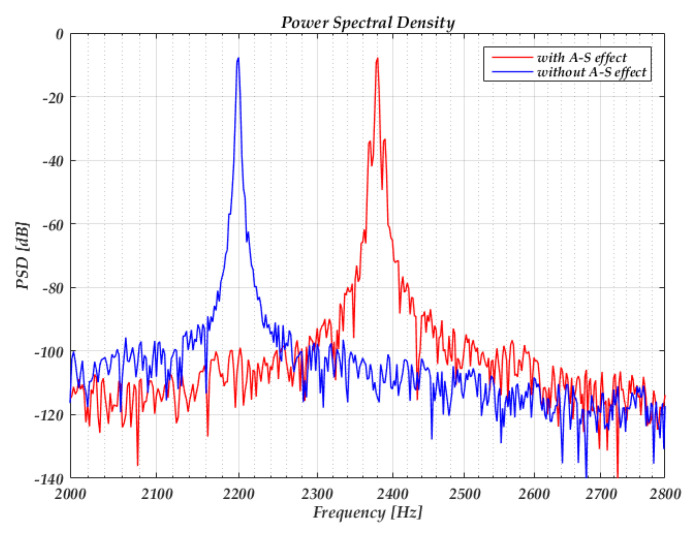
The power spectrum density of the output with the sine wave injected into the reference voltage.

Figure 10 shows the power spectrum density with and without the A-S effect when injecting the voltage sine wave into the reference voltage. In this simulation, the reference voltage is set to 0.5 V, a sine voltage with an amplitude of 2 mV and a frequency of 10 Hz is injected into the reference voltage. When we set *k*_2_ = 0, the sideband represents the amplitude modulation effect caused by the injecting voltage, the sideband power relative to the carrier is nearly −55 dB. When we set *k*_2_ = 4 × 10^12^, the sideband power relative to the carrier caused by the A-S effect is nearly −25 dB, which is well-suited with the predicted result of −23 dB according to Equation (49).

**Figure 11:**
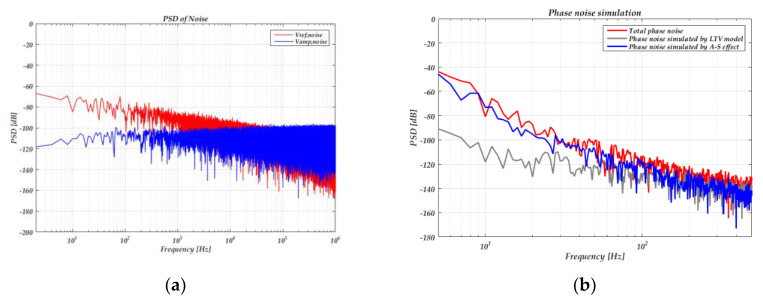
(**a**) Power spectrum density of the voltage noise added on the drive voltage and reference voltage; (**b**) phase noise simulation results.

Figure 11 shows the simulated phase noise. The power spectrum density of voltage noise added on the drive voltage and reference voltage are shown in Figure 11a. We set *k*_2_ = 0 when simulating the LTV model and set *k*_2_ = 4 × 10^12^, *V_amp,noise_* = 0, *V_ref_* = 0.5 V when simulating the A-S effect. Figure 11b shows that the phase noise caused by the A-S effect is larger than the LTV model under the simulation parameters above.

## 6. Experimental Results

**Figure 12:**
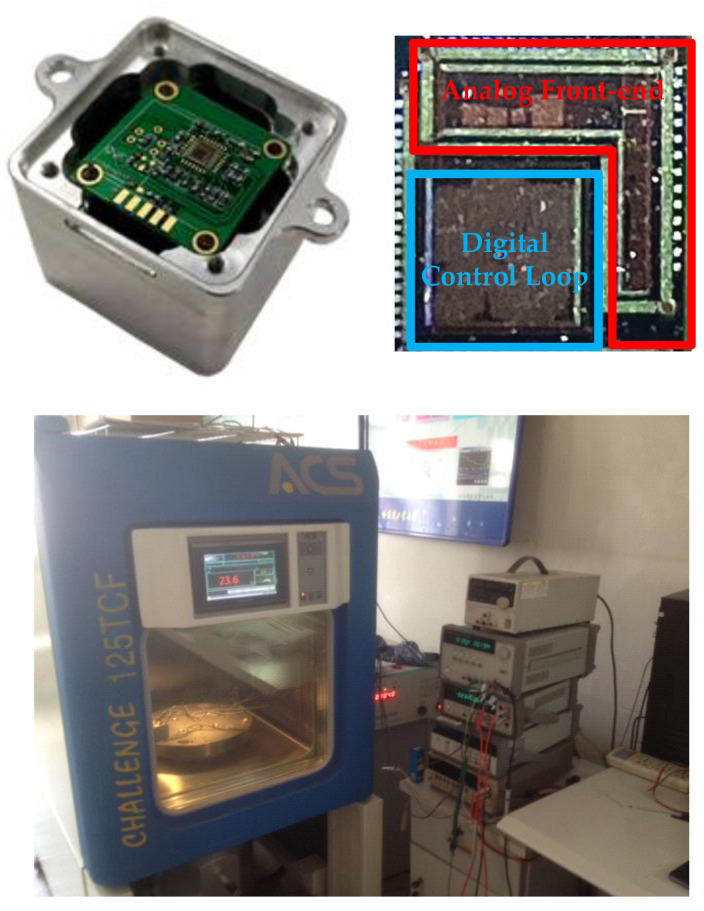
The interface integrated circuit and test system of the DRG.

**Figure 13:**
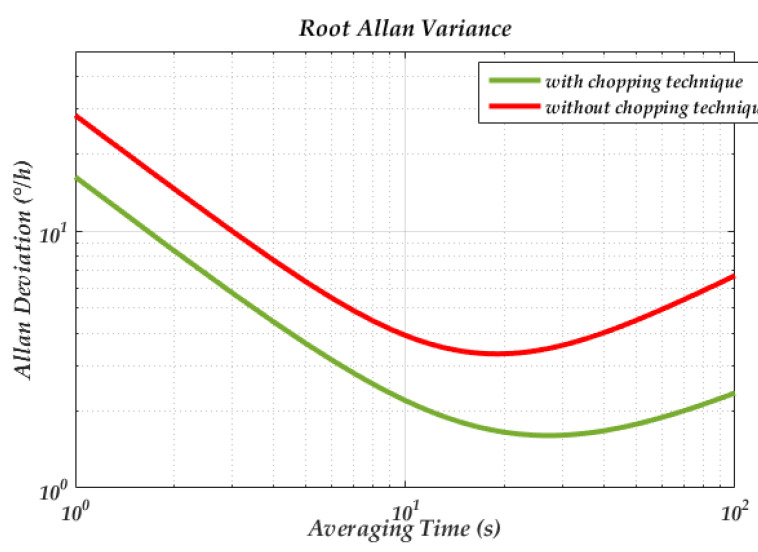
Standard Allen variance of the micro-electro-mechanical system (MEMS) DRG with different phase noises.

To verify the influence of the phase noise on the MEMS DRG system, a corresponding test circuit is used. The schematic of the loops is shown in Figure 2 and the picture of the experimental setup is shown in Figure 12. The circuit is fabricated in a standard 0.35 μm CMOS BCD process, which is combined with the analog front-end circuit and digital control loop. The chip area is 25 mm^2^ and the working current is 50 mA with a 5 V voltage supply. The input range and scale factor nonlinearity of the gyroscope are ±400°/s, 6.2 mV/°/s, and 0.035% respectively. More circuit design details and experimental data are described in our previous work [49]. In the experiment, we chose whether or not to use a chopping technique on the bandage reference in the drive loop (the reference voltage of the dive loop is generated by the reference voltage). Since the flicker noise can be significantly attenuate though the chopping technique [50], the phase noise caused by the A-S effect can be largely reduced. Figure 13 gives a standard Allen variance at 25 °C for the two cases. The experimental result shows that the bias instability is attenuated from 3.3°/h to 1.6°/h.

## 7. Discussion

Equation (53) gives the phase noise in drive loop. The first two terms in the equation is caused by the force noise and predicted by the proposed LTV model. Since the formula deduction is based on the ideal dynamical equation, even the flawless gyroscope may suffer from phase fluctuation caused by the force noise. The last term in Equation (53) is caused by the A-S effect and the main causation of this term is the nonlinearities of DRG. A recent study has found that electrostatic and capacitive nonlinearities are the main causation of nonlinearities and proposed a method to reduce them. Since the main purpose of this paper is to deduce the phase noise, the causation of the nonlinearity itself is not discussed here.

Equation (68) gives the impact of phase noise on DRG, which shows the phase noise in the drive loop may be transferred into low frequency noise after demodulation and deteriorate the bias stability. Since *Φ_xy_* in the equation is easily influenced by environmental change, a strict force-to-rebalance control loop, quadrature control loop, and phase adjustment circuit is needed.

The impact of the phase noise is derived from the demodulation aspect. Moreover, phase fluctuation (or frequency drift) in the drive loop may also affect the mode-matching condition of the DRG and deteriorate mechanical sensitivity dramatically. This impact needs to be further studied.

## 8. Conclusions

In this paper, a proposed model has been established to evaluate the phase noise in the drive loop of MEMS DRG, which combined the proposed LTV model with the A-S effect. Different from a traditional analysis, the LTV model is established in the mechanical domain, which gains more physical insight into the origin of phase noise. The impact of the phase noise on DRG performance is also derived, which shows that the phase noise in the drive loop together with non-ideal factors may deteriorate the gyroscope performance. Some design guides, both from a mechanical structural aspect and control loop aspect, are proposed to minimize the phase noise and its impact. Experimental results also show that, by reducing the phase noise in drive loop, the performance of DRG is optimized.

## Figures and Tables

**Table 1:** Comparison between the mechanical and electrical model.

Mechanical Domain Model	Electrical Model
$m_{x}\frac{d^{2}v_{x}}{dt^{2}}+D_{xx}\frac{dv_{x}}{dt}+k_{xx}v_{x}=\frac{dF_{x}}{dt}$	$L\frac{d^{2}i}{dt^{2}}+R\frac{di}{dt}+\frac{i}{C}=\frac{du_{d}}{dt}$
Mass *m_x_*	Inductance *L*
Damping coefficient *D_xx_*	Resistance *R*
Elasticity coefficient *k_xx_*	Capacitance *C*
Drive force *F_x_*	Drive voltage *u_d_*
Elastic force *F_k_*	Voltage on Capacitance *V_C_*
Frictional force *F_D_*	Voltage on Resistance *V_R_*
Resultant force *F_m_*	Voltage on Inductance *V_L_*
Velocity *v_x_*	Current *i*
Injected impulse Δ*J = F_inj_*Δ*t*	Injected flux linkage Δ*ψ = u_inj_*Δ*t*

**Table 2:** Parameters for the simulation.

Parameter	Value	Unit
*m_x_*	2.54 × 10^−6^	kg
*D_xx_*	2.2 × 10^−8^	N/m/s
*k_xx_*	485	N/m
*k*_2_	4 × 10^12^	N/m^3^
*k_F/V_*	5 × 10^−5^	N/V
*k_i/v_*	3 × 10^−6^	A/m/s
*R_V/i_*	3.62 × 10^6^	Ω
*V_ref_*	1	V

## References

[1] Challoner A.D., Ge H.H., Liu J.Y. Boeing Disc Resonator Gyroscope. Proceedings of the 2014 IEEE/ION Position, Location and Navigation Symposium-PLANS 2014.

[2] Ge H.H., Liu J.Y., Buchanan B. Bias Self-calibration Techniques using Silicon Disc Resonator Gyroscope. Proceedings of the 2015 IEEE International Symposium on Inertial Sensors and Systems (ISISS) Proceedings.

[3] Uppalapati B., Ahamed M.J., Chodavarapu V.P. Design and Analysis of Wafer-level Vacuum-Encapsulated Disk Resonator Gyroscope using a Commercial MEMS Process. Proceedings of the 2017 IEEE National Aerospace and Electronics Conference (NAECON).

[4] Hamelin B., Yang J., Daruwalla A., Wen H., Ayazi F. (2019). Monocrystalline Silicon Carbide Disk Resonators on Phononic Crystals with Ultra-Low Dissipation Bulk Acoustic Wave Modes. Sci. Rep..

[5] Li Q.S., Xiao D.B., Zhou X., Xu Y., Zhuo M., Hou Z.G., He K.X., Zhang Y.M., Wu X.Z. (2018). 0.04 degree-per-hour MEMS disk resonator gyroscope with high-quality factor (510 k) and long decaying time constant (74.9 s). Microsyst. Nanoeng..

[6] Rozelle D.M. (2009). The Hemispherical Resonator Gyro: From Wineglass To the Planets. Adv. Astronaut. Sci.

[7] Ahn C.H., Nitzan S., Ng E.J., Hong V.A., Yang Y., Kimbrell T., Horsley D.A., Kenny T.W. (2014). Encapsulated high frequency (235 kHz), high-Q (100 k) disk resonator gyroscope with electrostatic parametric pump. Appl. Phys. Lett..

[8] Gerrard D.D., Ahn C.H., Flader I.B., Chen Y.H., Ng E.J., Yang Y.S., Kenny T.W. Q-Factor Optimization in Disk Resonator Gyroscopes Via Geometric Parameterization. Proceedings of the 2016 IEEE 29th International Conference on Micro Electro Mechanical Systems (MEMS).

[9] Mirjalili R., Wen H., Serrano D.E., Ayazi F. Substrate-Decoupled Silicon Disk Resonators Having Degenerate Gyroscopic Modes with Q in Excess of 1-Million. Proceedings of the 2015 Transducers-2015 18th International Conference on Solid-State Sensors, Actuators and Microsystems (TRANSDUCERS).

[10] Behbahani A.H., M’Closkey R.T. (2017). Frequency analysis of a uniform ring perturbed by point masses and springs. J. Sound Vib..

[11] Behbahani A.H., Kim D., Stupar P., DeNatale J., M’Closkey R.T. (2017). Tailored Etch Profiles for Wafer-Level Frequency Tuning of Axisymmetric Resonators. J. Microelectromech. Syst..

[12] Xiao D., Yu D., Zhou X., Hou Z., He H., Wu X. (2017). Frequency Tuning of a Disk Resonator Gyroscope via Stiffness Perturbation. IEEE Sens. J..

[13] Schwartz D.M., Kim D., Stupar P., DeNatale J., M’Closkey R.T. (2015). Modal Parameter Tuning of an Axisymmetric Resonator via Mass Perturbation. J. Microelectromech. Syst..

[14] Prikhodko I.P., Gregory J.A., Clark W.A., Geen J.A., Judy M.W., Ahn C.H., Kenny T.W. Mode-Matched MEMS Coriolis Vibratory Gyroscopes: Myth or Reality?. Proceedings of the 2016 IEEE/ION Position, Location and Navigation Symposium (PLANS).

[15] Kim D., M’Closkey R. A MEM Vibratory Gyro with Mode-Matching Achieved by Resonator Mass Loading. Proceedings of the 2014 IEEE/ION Position, Location and Navigation Symposium-PLANS.

[16] Hegazi E., Abidi A.A. (2003). Varactor characteristics, oscillator tuning curves, and AM-FM conversion. IEEE J. Solid-St Circ..

[17] Rael J.J., Abidi A.A. Physical processes of phase noise in differential LC oscillators. Proceedings of the IEEE 2000 Custom Integrated Circuits Conference.

[18] Levantino S., Samori C., Bonfanti A., Gierkink S.L.J., Lacaita A.L., Boccuzzi V. (2002). Frequency dependence on bias current in 5-GHz CMOS VCOs: Impact on tuning range and flicker noise upconversion. IEEE J. Solid State Circuits.

[19] Nguyen C.T.C. (2007). MEMS technology for timing and frequency control. IEEE Trans. Ultrason. Ferroelectr. Freq. Control.

[20] Coram G.J. (2001). A simple 2-D oscillator to determine the correct decomposition of perturbations into amplitude and phase noise. IEEE Trans. Circuits Syst. I.

[21] Leeson D.B. (1966). A simple model of feedback oscillator noise spectrum. Proc. IEEE.

[22] Hajimiri A., Lee T.H. (1998). A general theory of phase noise in electrical oscillators. IEEE J. Solid State Circuits.

[23] Kaajakari V., Koskinen J.K., Mattila T. (2005). Phase noise in capacitively coupled micromechanical oscillators. IEEE Trans. Ultrason. Ferroelectr. Freq..

[24] Pardo M., Sorenson L., Ayazi F. A Phase-Noise Model for Nonlinear Piezoelectrically-Actuated MEMS Oscillators. Proceedings of the 2011 IEEE International Symposium of Circuits and Systems (ISCAS).

[25] Kaertner F.X. (1990). Analysis of White and F-Alpha Noise in Oscillators. Int. J. Circuit. Theory Appl..

[26] Razavi B. (1996). A study of phase noise in CMOS oscillators. IEEE J. Solid State Circuits.

[27] Demir A., Mehrotra A., Roychowdhury J. (2000). Phase noise in oscillators: A unifying theory and numerical methods for characterization. IEEE Trans. Circuits Syst. I Fundam. Theory Appl..

[28] Ward P., Duwel A. (2011). Oscillator Phase Noise: Systematic Construction of an Analytical Model Encompassing Nonlinearity. IEEE Trans. Ultrason. Ferroelectr. Freq..

[29] Imani A., Hashemi H. (2012). Analysis and Design of Low Phase-Noise Oscillators With Nonlinear Resonators. IEEE Trans. Microw Theory.

[30] Pardo M., Sorenson L., Pan W., Ayazi F. Phase Noise Shaping Via Forced Nonlinearity in Piezoelectrically Actuated Silicon Micromechanical Oscillators. Proceedings of the 2011 IEEE 24th International Conference on Micro Electro. Mechanical Systems (MEMS).

[31] He L., Xu Y.P., Palaniapan M. (2010). A State-Space Phase-Noise Model for Nonlinear MEMS Oscillators Employing Automatic Amplitude Control. IEEE Trans. Circuits Syst. I.

[32] Zhao J., Zhao Y., Wang X., Xia G.M., Qiu A.P., Su Y., Xu Y.P. (2016). A System Decomposition Model for Phase Noise in Silicon Oscillating Accelerometers. IEEE Sens. J..

[33] Agrawal D.K., Seshia A.A. (2014). An Analytical Formulation for Phase Noise in MEMS Oscillators. IEEE Trans. Ultrason. Ferroelectr. Freq..

[34] Nayfeh A.H., Younis M.I., Abdel-Rahman E.M. (2005). Reduced-order models for MEMS applications. Nonlinear Dyn..

[35] Younis M.I., Nayfeh A.H. (2003). A study of the nonlinear response of a resonant microbeam to an electric actuation. Nonlinear Dyn..

[36] Chorsi M.T., Chorsi H.T. (2018). Modeling and analysis of MEMS disk resonators. Microsyst. Technol..

[37] Chorsi M.T., Chorsi H.T., Gedney S.D. (2017). Radial-contour mode microring resonators: Nonlinear dynamics. Int. J. Mech. Sci..

[38] Li Q., Xiao D., Xu Y., Zhuo M., Zhou X., Zhang Y., Yu L., Wu X. (2020). Nonlinearity Reduction in Disk Resonator Gyroscopes Based on the Vibration Amplification Effect. IEEE Trans. Ind. Electron..

[39] Agrawal D.K., Woodhouse J., Seshia A.A. (2013). Modeling Nonlinearities in MEMS Oscillators. IEEE Trans. Ultrason. Ferroelectr. Freq..

[40] Wang G.S., Liu X.W. (2014). The Analysis of the Phase Noise of the Closed-Loop Driver Circuit in Micromechanical Gyroscope Based on the Phase-Locked Principle. Adv. Mat. Res..

[41] Phani A.S., Seshia A.A., Palaniapan M., Howe R.T., Yasaitis J.A. (2006). Modal coupling in micromechanical vibratory rate gyroscopes. IEEE Sens. J..

[42] (2004). IEEE Standard Specification Format Guide and Test Procedure for Coriolis Vibratory Gyros.

[43] Zhou X., Xiao D.B., Li Q.S., Hou Z.Q., He K.X., Chen Z.H., Wu Y.L., Wu X.Z. (2018). Decaying Time Constant Enhanced MEMS Disk Resonator for High Precision Gyroscopic Application. IEEE-ASME Trans. Mech..

[44] Chen F., Li X., Kraft M. (2016). Electromechanical Sigma–Delta Modulators (ΣΔM) Force Feedback Interfaces for Capacitive MEMS Inertial Sensors: A Review. IEEE Sens. J..

[45] Newland D.E. (1990). Mechanical Vibration Analysis and Computation. J. Acoust Soc. Am..

[46] Saukoski M., Aaltonen L., Halonen K.A.I. (2007). Zero-rate output and quadrature compensation in vibratory MEMS gyroscopes. IEEE Sens. J..

[47] Ismail A., Ashraf K., Metawea A., Mostfa I., Saeed A., Helal E., Essawy M., Abdelazim M., Ibrahim M., Raafat R. A High-Performance Self-clocked Digital-Output Quartz Gyroscope. Proceedings of the 2015 IEEE Sensors.

[48] Omar A., Elshennawy A., AbdelAzim M., Ismail A.H. Analyzing the Impact of Phase Errors in Quadrature Cancellation Techniques for MEMS Capacitive Gyroscopes. Proceedings of the 2018 IEEE Sensor.

[49] Wang Y.H., Fu Q., Zhang Y.F., Zhang W.B., Chen D.L., Yin L., Liu X.W. (2020). A Digital Closed-Loop Sense MEMS Disk Resonator Gyroscope Circuit Design Based on Integrated Analog Front-end. Sensors.

[50] Enz C.C., Temes G.C. (1996). Circuit techniques for reducing the effects of op-amp imperfections: Autozeroing, correlated double sampling, and chopper stabilization. Proc. IEEE.

